# Impact of low-frequency electromagnetic fields on DNA instability caused by environmentally and occupationally relevant chemicals in lymphocytes of donors with different demographic characteristics

**DOI:** 10.1007/s00204-026-04509-6

**Published:** 2026-07-14

**Authors:** Miroslav Mišík, Michael Kundi, Franziska Ferk, Hans-Peter Hutter, Armen Nersesyan, Michael Grusch, Siegfried Knasmueller

**Affiliations:** 1 Center for Cancer Research, Medical University of Vienna, Borschkegasse 8a, 1090 Vienna, Austria; 2 Department of Environmental Health, Center for Public Health, Medical University of Vienna, Vienna, Austria

**Keywords:** Low frequency electromagnetic radiation, 50 Hz, Genotoxicity, Comet assay, Co-exposure, Heavy metals

## Abstract

Aim of this study was to find out if demographic factors (age and body weight) have an impact on the sensitivity of peripheral lymphocytes towards DNA damage caused by extremely low frequency electromagnetic fields (ELF-EMF), to which humans are exposed worldwide. Cells from three different groups (young normal-weight, young obese, and old normal-weight individuals) were treated with different intensities of ELF-EMF (50 Hz, 50, 200 and 1000 µT) in combination with genotoxic agents, which cause damage to the genetic material via different molecular mechanisms. BPDE (benzo[a]pyrene diol epoxide) is a metabolite of the polycyclic aromatic hydrocarbon benzo(a)pyrene (B(a)P); 4-nitroquinoline oxide (4NQO) is a UV-mimetic agent; the metal compounds NiCl_2_ and CrO_3_ induce cross-links and oxidative damage. The cells were treated with radiation in combination with the different chemicals (simultaneous and sequential exposure), subsequently, DNA damage was monitored using single-cell gel electrophoresis (SCGE) experiments. We found no evidence for induction of genetic damage by the radiation in cells from the different groups. However, a decrease of damage was observed in combined experiments with NiCl_2_, and an increase of DNA migration was detected in some experiments with BPDE. These findings indicate that ELF-EMF does not cause DNA damage in lymphocytes regardless of the age and body weight of the donors. Furthermore, our results show that the radiation has an impact on the extent of DNA damage caused by specific environmentally and occupationally relevant exposures and may increase the genotoxic effects of polycyclic aromatic hydrocarbons.

**Supplementary Information:**

The online version contains supplementary material available at https://doi.org/10.1007/s00204-026-04509-6.

## Introduction

Humans are exposed worldwide to extremely low-frequency electromagnetic fields (ELF-EMF), the most common sources being 50 Hz fields generated by high-voltage power transmission lines, electrical power distribution systems, and household appliances (Baaken et al. 2020; Maffei 2022). High occupational expsure levels were found in workers from electric power plants and at specific industrial production sites (Gobba et al. 2004).

The health effects of ELF-EMF have been debated for decades. In 1979, a study reported increased risks of leukemia in children living in areas with high magnetic flux densities (Wertheimer and Leeper 1979). Based on epidemiological evidence suggesting a link between ELF-EMF exposure and childhood leukemia, as well as other cancers, the International Agency for Research on Cancer (IARC) classified ELF-EMF as a Group 2B carcinogen ("possibly carcinogenic to humans") in 2002 (IARC 2002). Subsequent evaluations, including a study by Schüz et al. (2016), concluded that the available evidence is consistent with possible carcinogenicity in humans. Subsequently a large-scale study involving over 7,000 animals exposed to 50 Hz magnetic fields for their entire lifespan, combined with γ-radiation or chronic formaldehyde administration, reported a significant increase in malignant tumors in Sprague–Dawley rats (Soffritti and Giuliani 2019).

Genetic alterations are a hallmark of human cancer (Hanahan and Weinberg 2011). Therefore, a large number of studies was realized which concerned the effects of ELF-EMF on DNA stability. Vijayalaxmi and Prihoda reviewed over 200 publications on this topic, and found in many publications methodological limitations and stated that lower-quality experiments were more likely to report positive results (Vijayalaxmi and Prihoda 2018).

We used in the present study an experimental model which we developed initially for experiments with high frequency fields that are characteristic for mobile phone communication (Misik et al. 2023a) to address two relevant questions which have not been studied in earlier investigations. (i) Do demographic factors (age, body weight) have an impact on the sensitivity of lymphocytes towards low frequency electromagnetic fields (50 Hz) which are characteristic for power lines and household electrical appliances? (ii) Do ELF-EMF have an impact on genetic damage caused by occupationally relevant exposures to DNA damaging carcinogens? It is known from earlier studies that very high BMI values and age alter the activity of DNA repair systems and lead to release of reactive oxygen species that cause damage of the genetic material (Azqueta et al. 2020; Setayesh et al. 2018). All experiments were realized in agreement with the quality criteria defined by Vijayalaxmi and Prihoda (2018). The extent of DNA damage was measured in single cell gel electrophoresis (SCGE) assays which are based on the determination of DNA migration in an electric field (Collins et al. 2023). This procedure detects single and double strand breaks and apurinic sites and is increasingly used in genetic toxicology (Cordelli et al. 2021). In the last years, standardized protocols have been published (Collins et al. 2023) which were followed in the present study.

The combined treatment experiments were performed with chemicals which cause different forms of primary DNA damage which are relevant for environmental and occupational exposure. 4-Nitoquinoline-N-oxide (4-NQO) causes base dimers that are induced by UV light (Wang et al. 2016). Benzo(a)pyrene-diol epoxide (BPDE) is the most relevant metabolite of benzo(a)pyrene (B(a)P, a representative of polycyclic aromatic hydrocarbons (PAHs). Humans are exposed to this group of carcinogens at different workplaces (e.g. coke ovens, metal production) and via air pollution (IARC 2012). Carcinogenic representatives of this group cause bulky DNA adducts and, as a consequence mutations at the gene and chromosomal level (Bausinger et al. 2016; Piberger et al. 2018), Nickel chloride (NiCl_2_) and chromium trioxide (CrO_3_) cause (like other metals) DNA-DNA crosslinks as well as DNA–protein crosslinks and generate DNA reactive oxygen species (Guo et al. 2019; O’Brien et al. 2003). Different occupational groups are exposed to these metals, for example electroplaters, and workers at production sites of alloys (IARC 2012; Wultsch et al. 2024). It is known that exposures of humans to these metals increase their cancer risk (for reviews see IARC 2002, 2012)).

## Materials and methods

### Chemicals and media

Chemicals (Triton X-100, Trizma base, NaOH, dimethylsulfoxide (DMSO), propidium iodide, EDTA, Roswell Park Memorial Institute 1640 Medium (RPMI), PBS, 4NQO, NiCl_2_ and CrO_3_) and foetal bovine serum (FBS) were from Sigma-Aldrich (Steinheim, Germany). Low melting point agarose (LMPA) was from Gibco (Paisley, UK), normal melting point agarose (NMPA) came from Serva (Heidelberg, Germany). A. Seidel (Biochemisches Institut für Umwelt-Karzinogene, Gernot Grimmer Stiftung, Germany) provided BPDE.

Stock solutions of 4NQO and BPDE were prepared with pure DMSO and were diluted with fresh RPMI medium. The metal compounds were dissolved in aqueous medium. All solutions were prepared fresh before each experiment.

### Participants, blood sampling and storage of the cells

The realisation of the study was approved by the Ethics Committee of the Medical University of Vienna (MUW, EK Nr: 2350/2019) and informed consent was obtained from all participants. The collection of blood samples and the demographic characteristics are described in detail in a previous publication concerning the effects of high frequency electromagnetic fields (Misik et al. 2023a).

Peripheral lymphocytes were collected from three groups namely young individuals with normal weights (YN, n = 11, age 24.8 ± 3.1; BMI 23.6 ± 2.9), young obese individuals (YO, n = 11, age 27.5 ± 2.7 BMI 34.4 ± 4.9) and older participants with normal weights (ON, n = 12, age 69.1 ± 7.6, BMI 24.6 ± 1.9).

Peripheral blood mononuclear cells (PBMC) were isolated by gradient centrifugation (800 g, 15 min, 16 °C) with Histopaque (Sigma–Aldrich, Steinheim, Germany). The pellets were suspended in 1000 µL RPMI and aliquoted in Biofreeze Medium (Biochrom AG, Berlin, Germany), frozen overnight at − 80 °C and stored in liquid nitrogen.

### ELF-EMF exposure unit

The exposure system (sXc-ELF) was purchased from the Foundation for Information Technologies in Society (IT'IS; Zurich, Switzerland). A detailed description of the unit can be found in a publication of Chen et al. (2021). Two chambers equipped with Helmholtz coils were placed inside an incubator (HeraCell 240 CO_2_ incubator, Kendro Laboratory, Germany), which maintained optimal conditions for cell cultivation (37 °C ± 0.1 °C, 5% CO_2_, and 95% humidity). A computer program was used to randomize cell exposure to ensure blinded conditions across all experimental series. Information regarding the treatment of the cultures was accessible only to an operator located at the IT'IS Foundation in Zurich and was disclosed after the evaluation of the cells was completed.

### Experiments concerning the impact of ELF-EMF on chemically induced DNA damage

**Fig. 1:**
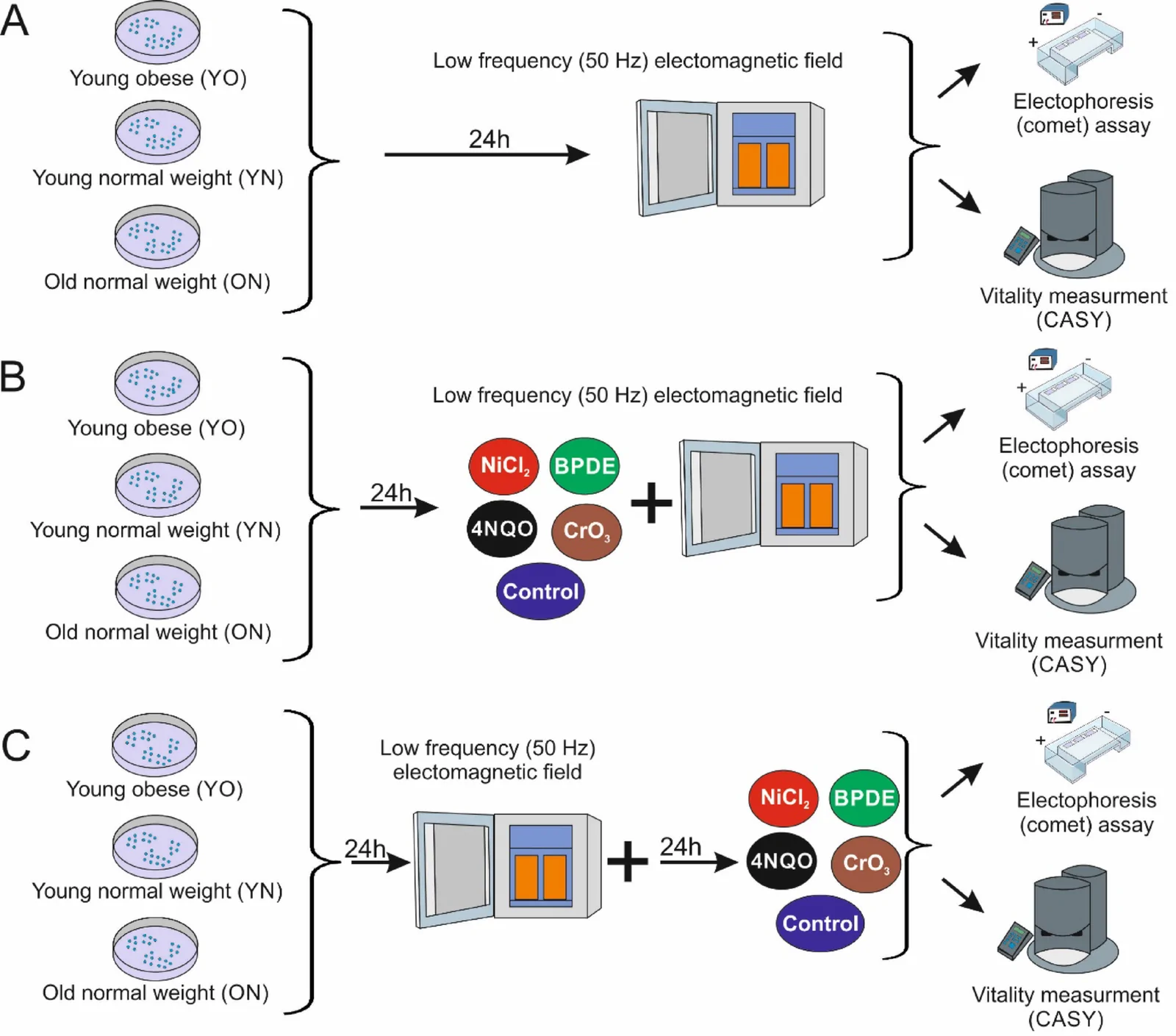
Treatment schemes of the cells. **A** Exposure to ELF-EMF only. **B** Simultaneous exposure to ELF-EMF and different chemicals for 24 h. **C** Sequential treatment with ELF-EMF for 24 h and with the chemicals (24 h)

The treatment schemes are shown in Fig. 1. Cells were irradiated and exposed either simultaneously or sequentially to the model mutagens. The optimal treatment conditions with the different chemicals were defined on the basis of dose response experiments (for details see supplementary Fig. 1). The following concentrations of the model mutagens were used in all combined treatment experiments: 4NQO (0.2 µM), BPDE (0.25 µM), CrO_3_ (30.0 µM), NiCl_2_ (450 µM).

In experiments with simultaneous treatment, PBMC from the three different groups were cultivated in 3.0 ml RPMI 10% serum in Petri dishes (35 mm ø Nunc, Roskilde, Denmark) for 24 h (75 × 10^3^ cells per ml). The cells (75 × 10^3^ cells per ml) were exposed to different ELF-EMF doses (50 Hz ELF-EMF, 0.0, 50 µT, 200 µT and 1000 µT) and in parallel to the chemicals for 24 h. 200 µT (the exposure guideline level for the general public defined by (ICNIRP 2010), 1000 µT is the limit value for occupational exposure in the European Union (directive 2013/35/EU). Three cultures per group were placed in an unexposed chamber (“sham-exposure”) and three were placed in an active chamber (with EMF exposure) (37 °C, 95% relative humidity, 5% CO_2_) in each experiment. In sequential treatment experiments, the cells were first exposed to different ELF-EMF flux densities (50 Hz ELF-EMF, 0.0, 50 µT, 200 µT and 1000 µT) for 24 h, followed by treatment with the chemicals for an additional 24 h.

### Determination of the viability of the cells

To avoid misinterpretations of comets we determined the viability of cells with a CASY counter (OMNI Life Science GmbH, Bremen, Germany) after the treatments (Koppen et al. 2017). Only cell cultures with a viability > 70% were evaluated in SCGE experiments.

### Single cell electrophoresis assay

The SCGE experiments were performed under standard alkaline conditions (Koppen et al. 2017). The cells were suspended in low melting point agarose (LMPA) and layered onto slides pre-coated with 1.5% normal melting point agarose (NMPA). Following lysis for at least 60 min (pH 10.0) and unwinding (30 min), electrophoresis was conducted (30 min, 300 mA, 0.8 V/cm, at 4 °C, pH > 13). Neutralization was carried out twice using double-distilled water (ddH2O) for 8 min each. The air-dried slides were stained with propidium iodide (20.0 μg/mL). The percentage of DNA in tail was determined by use of a computer-aided image analysis system (Comet IV, Perceptive Instruments Ltd., Haverhill, UK). From each culture, a minimum of 100 randomly distributed cells were evaluated (50 cells per slide). In every experiment, positive controls were included without additional chemical treatment (three cultures per experiment were exposed to 30 µM H_2_O_2_ for 5 min).

### Statistical methods

The distribution of DNA migration (%DNA in tail) was analysed in each slide and the median of the distribution of 50 cells per slide was taken as the parameter for further analysis.

The medians of the percentage DNA values were logit transformed to make variances homogeneous and to remove correlations between means and standard deviations as recommended (Misik et al. 2023b). The code numbers of the experiments were included in the model as random factor and the slides were nested within the experimental factors. Analysis of variance was applied (ANOVA) with the following fixed experimental factors: exposure (yes/no = sham), flux density: 50, 200, and 1000 µT and demographic groups: YO, YN and ON. Comparisons between treatments (sham versus exposed) within a demographic group were performed with sequential Sidak corrected linear contrasts. Normality of residues was checked in all experiments by Shapiro–Wilk tests and homogeneity of variances by Brown Forsythe tests. Dose response experiments with chemicals were analysed in a similar way with dose as the only experimental factor. Comparisons of the individual doses with the non-exposed (control groups) were done by Dunnett’s test. All analyses were performed with Stata 17.0 (StataCorp, College Station. TX, USA), the significance level was set to 5%.

## Results

**Fig. 2:**
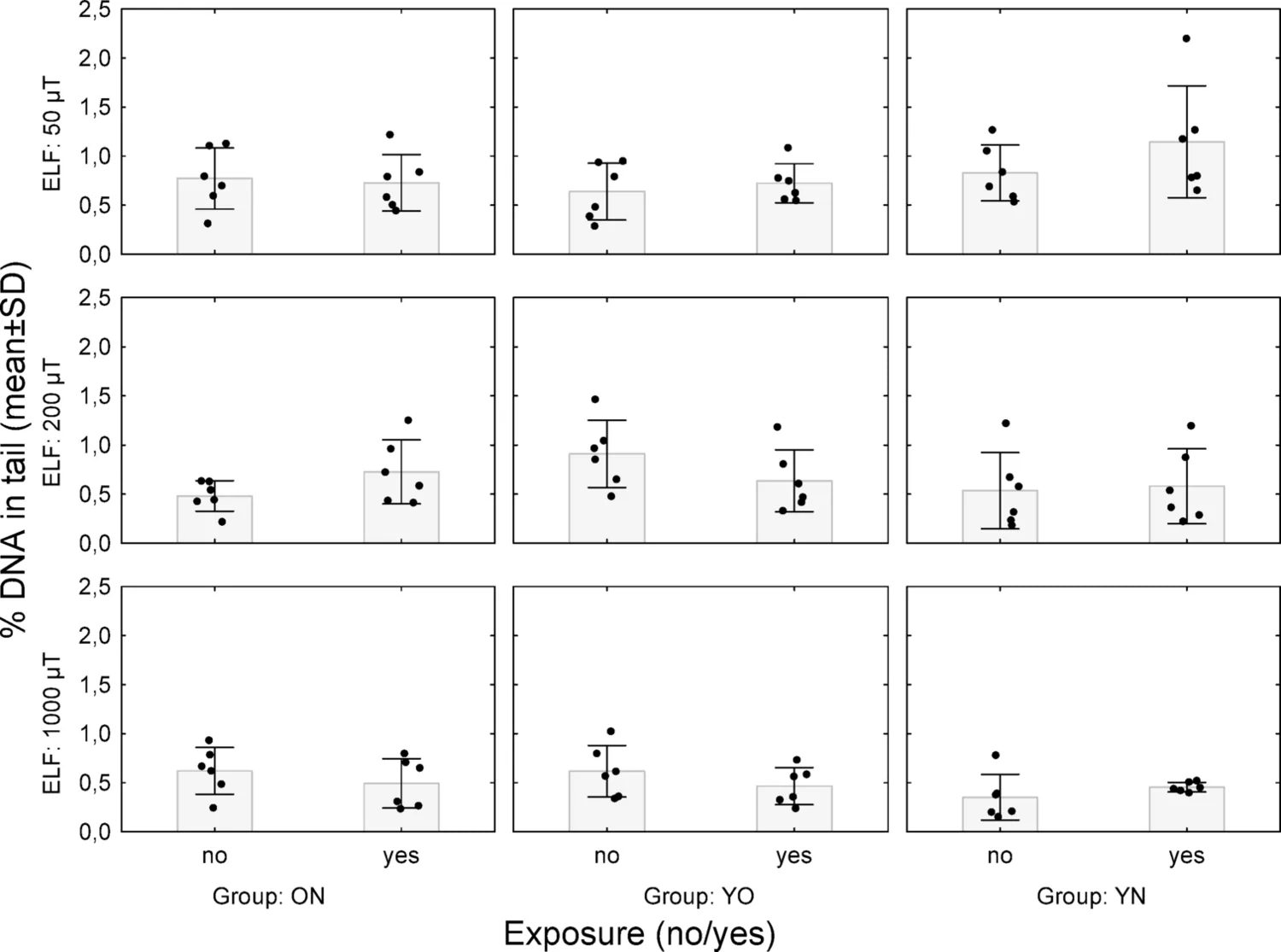
Impact of ELF-EMF on DNA stability in peripheral blood mononuclear cells from the three groups (young individuals with normal BMI (YN), old individuals with normal BMI (ON) and young individuals with increased BMI values (YO)). The lymphocytes were exposed to different flux densities (50 Hz, 50, 200 and 1000 µT). Per experimental point, 3 cultures were made. From each culture, a minimum of 100 randomly distributed cells was evaluated (50 cells per slide). Points indicate results from each slide. Bars show means ± SD., *p ≤ 0.05

Experiments, in which PBMCs from the three groups were exposed to different flux densities, demonstrated no impact of ELF-EMF on cell viability (data not shown). Furthermore, no significant differences were observed in the extent of DNA migration in single-cell gel electrophoresis assays in cells from the three groups (see Fig. 2).

In the combined treatment experiments, no impact of ELF-EMF fields on chemically induced DNA damage was observed when the cells were irradiated before exposure to the different model compounds (see Supplementary Fig. 2).

**Fig. 3:**
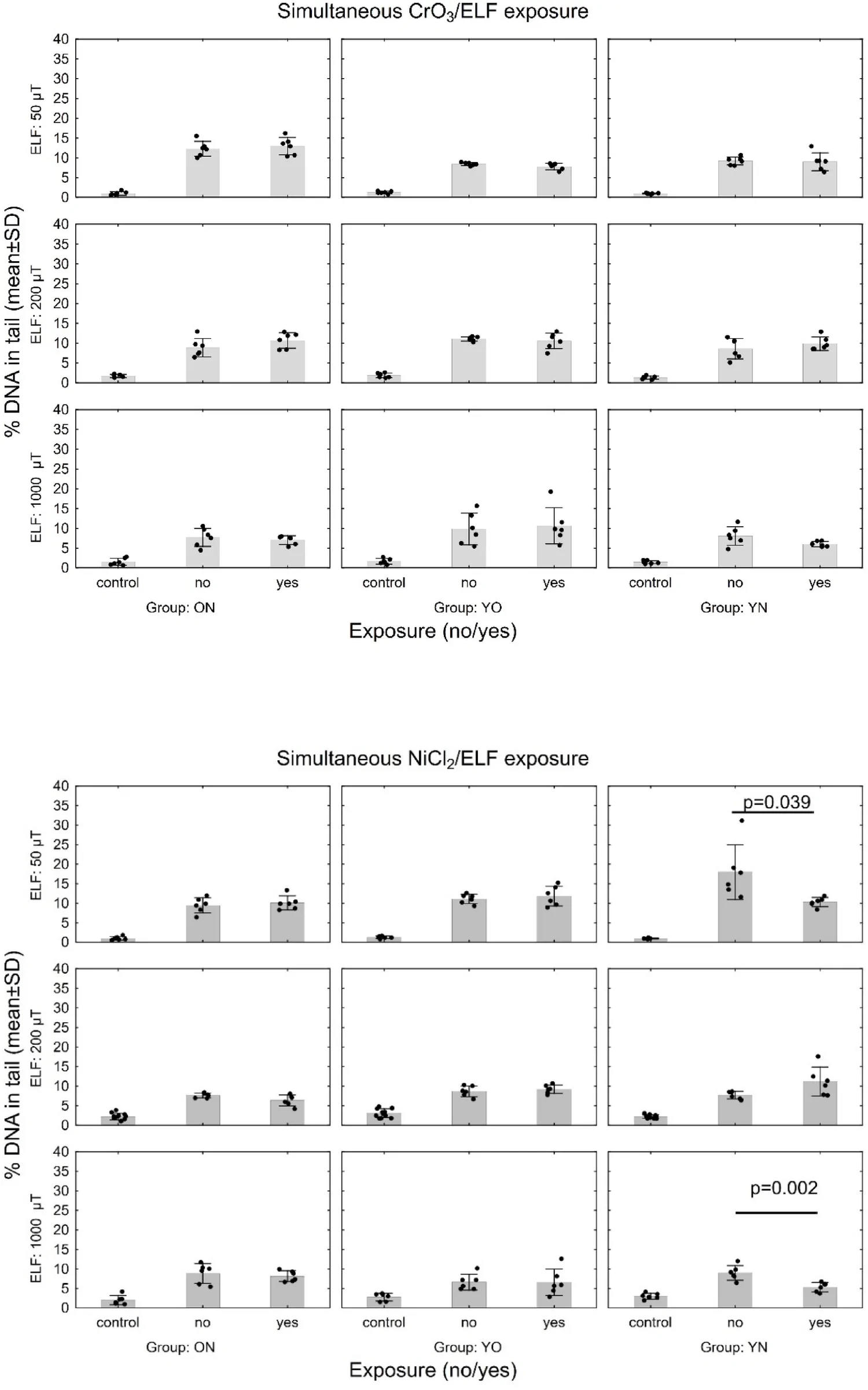
Impact of ELF-EMF on chemically induced DNA damage. The peripheral blood mononuclear cells were exposed to different flux densities (50 Hz, 50, 200 and 1000 µT) and to the test compounds (4NQO 0.2 µM, BPDE 0.25 µM, CrO_3_ 30.0 µM, NiCl_2_ 450 µM) for 24 h. Per experimental point, 3 cultures were made. From each culture, a minimum of 100 randomly distributed cells was evaluated (50 cells per slide). Points indicate results from each slide. Bars show means ± S.D, *p ≤ 0.05

The findings of the simultaneous exposure experiments (radiation and chemicals) are summarized in Fig. 3. In experiments with 4NQO, the extent of DNA migration was reduced when cells from young participants with normal weights were exposed simultaneously to ELF-EMF fields. This effect was observed at 200 µT and 1000 µT but did not reach statistical significance, however, a significant linear trend was detected across flux densities ranging between 50 and 1000 µT (*p* = 0.012). A similar phenomenon was noted in experiments with NiCl_2_, i.e. ELF-EMF led to a reduction of chemically induced comet formation. This effect was significant in experiments with 50 and 1000 µT. No clear alterations of the extent of DNA migration were observed in experimental series with cells from the other demographic groups.

A further relevant observation is the significant increase of DNA damage (by 64%) in cells from the YN group after treatment with BPDE, which was observed after simultaneous exposure to the highest intensity (1000 µM). It is notable that we found also an increase of DNA damage in cells from the YO group under identical conditions with 200 and 1000 µT, however this effect did not reach significance.

## Discussion

As mentioned in the introduction, the DNA damaging effects of ELF-EMF are controversially discussed (IARC 2002; Vijayalaxmi and Prihoda 2018). The clarification of this issue is also relevant for the assessment of potential adverse health effects of this form of radiation in humans. Aim of this study was to find out if exposure to 50 Hz ELF-EMF which is characteristic for power lines and household electrical appliances causes damage in PBMC and if demographic parameters (age, body weight) have an impact on the sensitivity of the cells. Furthermore, we investigated if this form of radiation affects the extent of DNA damage caused by chemicals which reflect exposures in occupational settings. To clarify these questions, we used an experimental model which was used successfully in an earlier study with high frequency electromagnetic fields that are employed for mobile communication (UMTS signal 1950 MHz), (Misik et al. 2023a).

We observed no differences of DNA migration in experiments without ELF exposure and chemical treatments (Fig. 2). Some earlier studies, reported an age-related increase in comet formation in PBMCs. (Humphreys et al. 2007; Soares et al. 2014), furthermore, elevated DNA damage was found in overweight subjects (Ferk et al. 2023; Setayesh et al. 2018). However, these differences were only significant when groups were analysed, which differed strongly in regard to age and body weights. Since our study focused on occupational exposures, all participants fell within a relatively normal range characteristic for workers and the extent of DNA migration in all three groups was consistent with the range observed in previous studies with the general population (Milić et al. 2021).

The four model compounds used in this study induce primary genetic damage via different molecular mechanisms. All agents caused positive results in genotoxicity test systems involving mammalian cells (Andersson et al. 2003; Bausinger et al. 2016; Chen et al. 2003; IARC 2012) and it is well documented that the molecular mechanisms which cause damage of the genetic material in cultured cells are also relevant for the exposure of humans (EFSA 2011). The ranking order of activity observed in a previous investigation was confirmed in the present study (4NQO > BPDE > CrO_3_ > NiCl_2_) was identical to that, which we observed in earlier SCGE experiments.

Exposure of cells from the different demographic groups to ELF-EMF did not cause damage of the genetic material (Fig. 2). These findings indicate that the age and the body weight of the donors had no impact on the sensitivity of the cells. The results of earlier studies concerning ELF fields are described in comprehensive reviews (Falone et al. 2018; IARC 2002; Tian et al. 2023) and the findings controversial. Most studies used human-derived cell lines and highly variable exposure conditions. Earlier in vitro studies with PBMCs concerning chromosomal damage and formation of micronuclei yielded mainly negative results. (IARC 2002; Testa et al. 2004; Verheyen et al. 2003). However, significant induction of DNA damage was observed in two studies with lymphocytes from individuals which were occupationally exposed to high intensities of ELF-EMF in SCGE experiments (Hosseinabadi et al. 2019; Zendehdel et al. 2019), while a more recent study by Nguyen et al. (2024) failed to detect DNA damage as a consequence of exposure to 50 Hz fields. As mentioned above, the exposure of the cells to the radiation was (in contrast to our study) not well controlled in most investigations (Vijayalaxmi and Prihoda 2018).

We found in the present study limited evidence for synergistic effects between ELF-EMF and genotoxic chemicals. Findings of earlier investigations are summarized in a review of Falone et al. (2018). The studies were mainly realized with cytostatic drugs and other pharmaceuticals. Potentiation of genotoxic effects was seen, for example, in tests with methyl methanesulfonate and cisplatinum (Kesari et al. 2016; Koyama et al. 2008; Sanie-Jahromi et al. 2016). An older study with workers in a transformer factory exposed to low-frequency fields suggested that co-exposure to mineral oils may lead to increased chromosomal aberrations in peripheral lymphocytes (Skyberg et al. 2001). No other studies with chemically exposed workers have been published according to our knowledge.

Interestingly, we found in the experiments with NiCl_2_, (which causes DNA-DNA crosslinks and oxidative damage), a decrease of DNA damage after exposure of the cells to ELF-EMF (Fig. 3). The effect was not dose dependent and was observed after treatment of the cells with 50 and 1000 µT. A statistically significant declining trend of DNA damage with increasing flux densities was also observed with 4NQO in the same group (YN). In this context it is notable that protective effects of ELF-EMF were also reported in earlier experiments with H_2_O_2_, which causes oxidative damage and with cisplatinum, (Buldak et al. 2012), menadione (Luukkonen et al. 2017) and β-lapachone (Sanie-Jahromi et al. 2017). It was hypothesized that the protective effects may be caused by adaptive responses, for example by induction of DNA repair functions, which were also observed when cells are exposed to ionizing radiation (Guéguen et al. 2019).

In contrast to these observations, we found a significant increase of BPDE-induced DNA migration when cells of individuals from the YN group were treated with 1000 µT (Fig. 3). A moderate increase (albeit not significant) was also seen in this experimental series under identical conditions in cells from the YO group. BPDE is the most relevant carcinogenic metabolite of benzo(a)pyrene and causes, like other metabolites of PAHs, bulky DNA adducts (mainly with guanosine) which lead to chromosomal aberrations and base substitutions (Ewa and Danuta 2017; IARC 2012). In this context, it is notable that Simkó et al. (2001) reported more than 20 years ago, that 50 Hz fields, (1000 µT), increase micronucleus frequencies (which are indicative for chromosomal damage), in Syrian hamster embryo (SHE) cells, while no effect was seen in an in vivo study with rats in peripheral blood reticulocytes (Singh et al. 1997). Humans are exposed to PAHs in many different occupational settings (Louro et al. 2022), also grilled foods, tobacco smoke as well as fuel exhausts and industrial emissions contain representatives of this highly relevant groups of carcinogens (Ewa and Danuta 2017).

Overall, the results of the present study indicate that exposure to high intensities of ELF-EMF may increase the adverse health effects of PAHs, but further experimental work is needed to clarify this assumption. With all other occupationally relevant exposures, no evidence for potential negative consequences of ELF-EMF radiation was observed in the present study.

## Supplementary Information

Below is the link to the electronic supplementary material.

Supplementary Material 1.

## Data Availability

The data underlying this article will be shared on reasonable request to the corresponding author. Information on individual participants will not be made available.
