# Supplementary material for: Impact of low-frequency electromagnetic fields on DNA instability caused by environmentally and occupationally relevant chemicals in lymphocytes of donors with different demographic characteristics
Source: Arch Toxicol. 2026 Jul 14;100(12):5673–81. doi: 10.1007/s00204-026-04509-6 (PMC13748046; doi:10.1007/s00204-026-04509-6)
Supplement: Supplementary file 1 [file 204_2026_4509_MOESM1_ESM.docx]

|  |  |
| --- | --- |
|  |  |

Supplementary figure 1: Induction of DNA damage by the different model compounds. Peripheral blood mononuclear cells from YN individuals (young, with normal BMI values) were treated for 24 hours with different concentrations of the chemicals. Per experimental point, 3 cultures were made. From each culture, a minimum of 100 randomly distributed cells were evaluated (50 cells per slide). Points indicate results from individual slides. Bars in the figures show means ± S.D., *p≤0.05, **p≤0.01 and ***p≤0.001.

Supplementary figure 2: Impact of ELF-EMF on chemically induced DNA damage. The peripheral blood mononuclear cells were exposed to different flux densities (50 Hz, 50, 200 and 1000 µT) for 24 hours. Subsequently, they were treated with the model compounds (4NQO 0.2 µM, BPDE 0.25 µM, CrO_3_ 30.0 µM, NiCl_2_ 450 µM) for an identical time period. Per experimental point, 3 cultures were made. From each culture, a minimum of 100 randomly distributed cells was evaluated (50 cells per slide). Points indicate results from individual slide. Bars show means ± S.D., *p≤0.05.
